# Improving Safety of Preemptive Therapy with Oral Valganciclovir for Cytomegalovirus Infection after Allogeneic Hematopoietic Stem Cell Transplantation

**DOI:** 10.1155/2012/874601

**Published:** 2012-12-03

**Authors:** Corinna Barkam, Haytham Kamal, Elke Dammann, Helmut Diedrich, Stefanie Buchholz, Matthias Eder, Jürgen Krauter, Arnold Ganser, Michael Stadler

**Affiliations:** Department of Hematology, Hemostasis, Oncology, and Stem Cell Transplantation, Hannover Medical School, 30625 Hannover, Germany

## Abstract

Valganciclovir (VGC), an oral prodrug of ganciclovir (GCV), has been shown to clear cytomegalovirus (CMV) viremia in preemptive treatment of patients after allogeneic hematopoietic stem cell transplantation (alloHSCT), apparently without significant toxicity. Since VGC obviates hospitalization, it is increasingly being adopted, although not approved, in alloHSCT. When we retrospectively evaluated preemptive treatment with VGC versus GCV, foscarnet or cidofovir, in all 312 consecutive CMV viremias of 169 patients allotransplanted at our institution between 1996 and 2006, we found VGC more efficacious (79%) than non-VGC therapies (69%). The advantage of outpatient VGC, however, was outbalanced by more profound neutropenias (including two cases of agranulocytosis, one with graft loss) requiring subsequent prolonged rehospitalization. Thus, in a second, prospective cohort from 2007 to 2011 (all 202 consecutive CMV viremias of 118 yet older and sicker patients), we implemented twice weekly neutrophil monitoring during outpatient VGC treatment and avoided VGC maintenance therapy. While conserving efficacy (VGC 71%, non-VGC 72%), we could now demonstrate a reduced mean duration of hospitalization with VGC (9 days (0–66)) compared to non-VGC (25 days (0–115)), without any agranulocytosis episodes. We conclude that safe outpatient VGC therapy is possible in alloHSCT recipients, but requires frequent monitoring to prevent severe myelotoxicity.

## 1. Introduction

Although mortality from cytomegalovirus (CMV) infection after allogeneic hematopoietic stem cell transplantation (alloHSCT) has largely decreased with modern preemptive treatment, CMV viremias still contribute to significant morbidity and a considerable hospitalization burden for intravenous therapy with the standard first-line CMV drugs ganciclovir (GCV) and foscarnet (FCN).

Valganciclovir (VGC), an orally available prodrug hydrolyzed to GCV, with a tenfold bioavailability compared to oral GCV [1], has been licensed for therapy of CMV retinitis in HIV disease and for CMV prophylaxis after solid organ transplantation, but not after alloHSCT, due to concern about its myelotoxicity, especially in long-term application. However, VGC has enjoyed widespread off-label use thanks to its outpatient applicability and its excellent bioavailability even in patients with impaired resorption due to intestinal graft-versus-host disease [2–4]. Several smaller trials and one large study found high efficacy (73%–100%) of VGC in the preemptive setting after alloHSCT ([5–14]; see Table 1), comparing favourably with standard CMV drugs. However, these mostly retrospective studies with short follow up may have underestimated toxicity, although some of them reported up to 27% severe hematotoxicity, especially neutropenia.

Here, we present the largest single-center study to date on VGC in preemptive treatment of CMV viremias after alloHSCT, assessing the neutropenia risk of VGC in a well-sized retrospective cohort and evaluating a prospective surveillance strategy to improve safety in outpatient VGC therapy.

## 2. Patients and Methods

### 2.1. Study Design

This is a combined retrospective and prospective comparative cohort study, analyzing all consecutive CMV viremias after alloHSCT treated preemptively at our institution during the years 1996 through 2006 (retrospective cohort 1: 169 patients; 312 CMV viremias) as well as 2007 through 2011 (prospective cohort 2: 118 patients; 202 CMV viremias). Excluded were patients originally not allotransplanted at our institution and patients without details on CMV treatment at other hospitals. Data are as of July 1st, 2012, thus allowing for at least six months of follow up. Patient data evaluation was in complete concordance with the declaration of Helsinki. All patients had given written informed consent prior to transplantation; prior to preemptive CMV therapy, informed consent was again obtained.

### 2.2. Diagnosis and Preemptive Treatment of CMV Viremias

According to our institutional standard operating procedures, every patient after alloHSCT was followed weekly until immune reconstitution by CMV pp65 antigen testing or, during neutropenia, by CMV DNA PCR. Preemptive therapy was started when a significant increase of CMV pp65 antigen was noted (generally more than 5 per 400.000 leukocytes positive or CMV DNA PCR exceeding 10.000 copies per mL whole blood). Preemptive therapy of CMV viremias was selected according to the patients' toxicity profile: GCV in case of renal impairment, FCN if hematopoietic reconstitution was yet insufficient. Patients without hematopoietic or renal compromise were given the choice between intravenous GCV and oral VGC, after counseling about side effects of both drugs as well as about the off-label status of VGC. Standard doses of VGC (900 mg every 12 hours), GCV (5 mg per kg body weight every 12 hours), FCN (90 mg per kg body weight every 12 hours), or cidofovir (CDF; 5 mg per kg body weight once a week, but not as first-line therapy) were used with appropriate hydration and supportive measures and tailored according to renal function, as detailed in the manufacturers' descriptions. Initial preemptive therapy was given for at least two weeks (or changed to a second-line drug if viremia progressed); it was stopped after three consecutive negative CMV tests.

Patients were documented in the “VGC group” if they had received VGC at any time during a CMV viremia episode, if not, they belonged to the “non-VGC group.”

In cohort 1, maintenance therapy was used, mostly at half the initial dose, in patients with unfavourable CMV constellation (recipient positive/donor negative; R+/D−) and in patients with repeated CMV viremias. In cohort 2, maintenance treatment was avoided, whenever possible, in order to prevent profound neutropenias. Patients in cohort 2 who opted for outpatient VGC were instructed to have complete blood counts controlled twice a week for early detection of neutropenia.

### 2.3. Patient Cohorts

Both cohorts are described in detail in Table 2. In cohort 1, despite the retrospective study design, both the VGC and the non-VGC group were comparable for demographic, disease- and transplant-related aspects such as gender, diagnosis, and donor type. However, VGC patients of cohort 1 were generally older, had more advanced disease, showed more often the unfavourable CMV constellation R+/D−, and more of them had received reduced intensity conditioning and *in vivo* T cell depletion with antithymocyte globulin (ATG). In contrast, the VGC and the non-VGC groups of cohort 2 were much better balanced with respect to all demographic, disease- and transplant-related characteristics, even conditioning and *in vivo* T cell depletion, but especially age and disease status which were considerably less favourable in both groups of cohort 2 compared to cohort 1. Thus, a historical trend is evident towards alloHSCT in patients of ever increasing age and morbidity.

### 2.4. Statistics

Due to the retrospective design of cohort 1, statistics were primarily descriptive. Statistical comparisons were calculated from cross-tables employing two-sided Chi-square tests.

## 3. Results and Discussion

### 3.1. Efficacy of VGC in Preemptive Therapy of CMV Viremias

In cohort 1, the rate of CMV clearance was better for patients preemptively treated with VGC (79%) than with non-VGC therapies (69%), *P* = 0.04. In cohort 2, efficacy was similar in both groups: VGC cleared CMV viremia in 71%, non-VGC treatments in 72% (not significant). These results are in concordance with and confirm published studies (see Table 1).

### 3.2. Hospitalization Requirements for VGC versus Non-VGC Preemptive Therapies

As expected, hospitalization for preemptive therapy was lower in VGC-treated patients. However, in cohort 1, severe neutropenias in the VGC group accounted for prolonged subsequent rehospitalizations. Thus, when considering both initial preemptive therapy and later treatment of complications, there was no difference between the groups in mean total hospitalisation (cohort 1: VGC 8 days (0–257); non-VGC 10 days (0–89); not significant). Of course, as suggested by the wide range of hospitalization duration in the VGC group of cohort 1, few outliers with excessive subsequent rehospitalizations (see Section 3.3) were responsible for this statistical effect which, however, highlights well the potential danger from VGC treatment in terms of profound and prolonged myelotoxicity.

**Figure 1:**
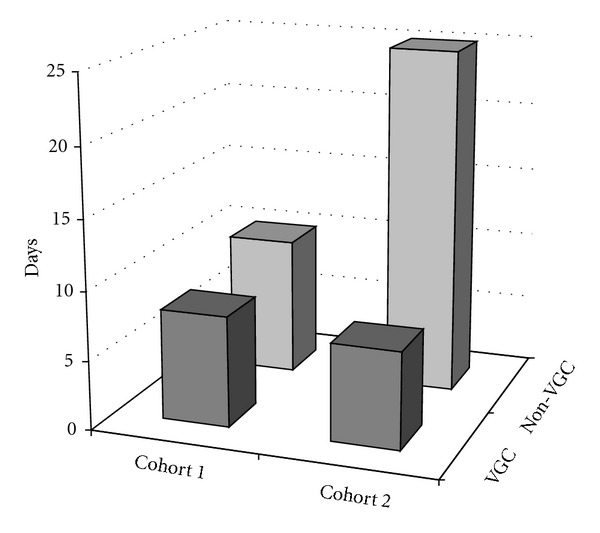
Mean total hospitalization for treatment of CMV viremia plus complications.

This observation led us to implement frequent neutrophil monitoring during outpatient VGC treatment in cohort 2. Here, likely as a consequence of our change in surveillance strategy, we were able to demonstrate a reduced mean duration of total hospitalization after VGC (9 days (0–66)) as compared to non-VGC therapies (25 days (0–115)), with a much narrower range in the VGC group (Figure 1).

Surprisingly, mean hospitalization in the non-VGC groups differed substantially between cohorts 1 and 2. A potential explanation for this may derive from the significant differences between both cohorts, especially regarding patient age, disease risk, and mismatched donors. Compared to cohort 1, where the non-VGC group enjoyed greatly favourable characteristics with respect to the VGC group, in cohort 2, both groups were strongly disadvantaged; thus, patients may have suffered more toxicities from non-VGC therapies, too. Other possible factors for differences between the cohorts include toxicities from concomitant medications which might have been unequally distributed in consecutive cohorts. Finally, the possibility should be considered that prevalent CMV strains were becoming increasingly resistant over time; thus, more patients might have required additional CMV therapies.

### 3.3. Myelotoxicity of VGC in Preemptive CMV Treatment after AlloHSCT

In cohort 1, although numerically comparable (11% in both the VGC and the non-VGC group), severe neutropenias were of greater clinical importance in the VGC group, compatible with a more profound and/or prolonged suppressive effect of VGC on granulopoiesis [2]. Especially, two cases of agranulocytosis occurred, which were confirmed by bone marrow biopsy. Whereas one patient recovered with granulocyte-colony stimulating factor after more than one month of hospitalization, the other patient additionally experienced a complete loss of his graft, eventually requiring retransplantation 6 months after preemptive VGC therapy; unfortunately, he died 4.5 months after the second transplant from infectious complications.

In cohort 2, neutropenias occurred in significant less patients of the VGC group (*n* = 9) than in patients of the non-VGC group (*n* = 37), *P* = 0.004. Conversely, the duration of neutropenias was not different (VGC: median 8 days, range 1–14; non-VGC: median 7 days, range 1–34; not significant). No cases of agranulocytosis or graft loss were observed in cohort 2.

Suppression of granulopoiesis was the most clinically relevant myelotoxicity of VGC encountered in our cohorts. When we compared frequencies of anemia, thrombocytopenia, red blood cell transfusion, and platelet substitution, we could not detect any significant differences in either cohort (data not shown).

### 3.4. Strengths and Limitations of the Study

The main limitations of this study are the retrospective design of cohort 1 and the difficulty to directly compare both cohorts due to the differences between them; however, the comparisons within each cohort still remain unchallenged. Another shortcoming is the nonrandomized treatment allocation, introducing a patient's bias with the patient's choice; however, this may better reflect real clinical practice in preemptive therapy of CMV viremias after alloHSCT.

Strengths of this study are its well-sized cohorts, making it the currently largest study with the longest follow up on VGC in the preemptive setting after alloHSCT. It is thus suited for the detection of rare, but severe, events and appropriate for assessment of a surveillance strategy to improve the safety of treatment with this widely used, but narrowly evaluated, yet unlicensed drug.

## 4. Conclusions

The present study combines adequately sized retrospective and prospective cohorts to examine the significant neutropenia risk of VGC for CMV viremia in the preemptive setting after alloHSCT. We show that outpatient VGC therapy can be safely applied in these patients, following a simple surveillance strategy with frequent neutrophil monitoring and using VGC maintenance therapy with utmost caution.

Further large, prospective studies on the use of VGC after alloHSCT are welcome.

## Figures and Tables

**Table 1:** Studies on VGC in the preemptive setting after alloHSCT.

Author year [ref.]	Design	*N* patients	VGC dose	VGC efficacy	Toxicity reported
van der Heiden et al. 2006 [5]	Retrospective VGC/GCV	34	1800 mg/d	80%	None
Ayala et al. 2006 [6]	Retrospective VGC mono	18	1800 mg/d × 14, → 900 mg/d × ≥7	93%	None
Busca et al. 2007 [7]	Retrospective VGC mono	15	1800 mg/d × 14, → 900 mg/d × 14	73%	27% severe hematotoxicity
Candoni et al. 2008 [8]	Retrospective VGC mono	30	1800 mg/d versus 900 mg/d	93% 87%	≤WHO °II
de la Cruz-Vicente et al. 2008 [9]	Prospective VGC/GCV	11	1800 mg/d × 14, → 900 mg/d × 14	100%	None
Wang et al. 2008 [10]	Retrospective VGC mono	19	1800 mg/d × 14, → 900 mg/d × 14	95%	None
Takenaka et al. 2009 [11]	Retrospective VGC mono	10	1800 mg/d × 21	90%	10% severe neutropenias
Saleh et al. 2010 [12]	Retrospective VGC mono	47	1800 mg/d ×≥7, → 900 mg/d ×≥7	97%	17% severe neutropenias
Palladino et al. 2010 [13]	Retrospective VGC mono	34	1800 mg/d versus 900 mg/d	100% 83%	None
Liu et al. 2010 [14]	Prospective VGC mono	54	1800 mg/d × 14, → 900 mg/d × 14	89%	None
Ruiz-Camps et al. 2011 [15]	Prospective VGC/GCV	166		91%	5% adverse events

**Table 2:** Patient cohorts of preemptive therapy for CMV viremias.

	Cohort 1 (1996–2006)			Cohort 2 (2007–2011)		
	VGC	Non-VGC	*P *	VGC	Non-VGC	*P *
*N* patients	79	90	—	48	70	—
*N* viremias	165	147	—	67	135	—
Gender						
Female	.44	.44	n.s.	.50	.47	n.s.
Male	.56	.56		.50	.53	
Age						
Median	50	43	<0.001	53	56	n.s.
Range	18–68	18–65		20–67	19–72	
Diagnosis						
Nonmalignant	.03	.00	n.s.	.03	.04	n.s.
Chronic malignancy	.24	.26		.17	.32	
Acute malignancy	.73	.74		.80	.64	
Disease risk						
Standard	.29	.47	<0.05	.11	.23	n.s.
Advanced	.71	.53		.89	.77	
Donor						
Matched related	.28	.43	n.s.	.25	.21	n.s.
Matched unrelated	.68	.52		.44	.53	
Mismatched	.04	.05		.31	.26	
CMV						
R−/D−, R−/D+, R+/D+	.51	.67	<0.05	.69	.70	n.s.
R+/D−	.49	.33		.31	.30	
Conditioning						
Reduced intensity	.65	.30	<0.001	.83	.79	n.s.
Myeloablative	.35	.70		.17	.21	
T cell depletion						
None	.11	.40		.06	.08	n.s.
*In vivo* (ATG)	.84	.44	<0.001	.94	.92	
*In vitro *	.05	.16		.00	.00	

## References

[1] Reusser P (2001). Oral valganciclovir: a new option for treatment of cytomegalovirus infection and disease in immunocompromised hosts. *Expert Opinion on Investigational Drugs*.

[2] Einsele H, Reusser P, Bornhäuser M (2006). Oral valganciclovir leads to higher exposure to ganciclovir than intravenous ganciclovir in patients following allogeneic stem cell transplantation. *Blood*.

[3] Winston DJ, Baden LR, Gabriel DA (2006). Pharmacokinetics of ganciclovir after oral valganciclovir versus intravenous ganciclovir in allogeneic stem cell transplant patients with graft-versus-host disease of the gastrointestinal tract. *Biology of Blood and Marrow Transplantation*.

[4] Lim ZY, Cook G, Johnson PR (2009). Results of a phase I/II british society of bone marrow transplantation study on PCR-based pre-emptive therapy with valganciclovir or ganciclovir for active CMV infection following alemtuzumab-based reduced intensity allogeneic stem cell transplantation. *Leukemia Research*.

[5] van der Heiden PLJ, Kalpoe JS, Barge RM, Willemze R, Kroes ACM, Schippers EF (2006). Oral valganciclovir as pre-emptive therapy has similar efficacy on cytomegalovirus DNA load reduction as intravenous ganciclovir in allogeneic stem cell transplantation recipients. *Bone Marrow Transplantation*.

[6] Ayala E, Greene J, Sandin R (2006). Valganciclovir is safe and effective as pre-emptive therapy for CMV infection in allogeneic hematopoietic stem cell transplantation. *Bone Marrow Transplantation*.

[7] Busca A, de Fabritiis P, Ghisetti V (2007). Oral valganciclovir as preemptive therapy for cytomegalovirus infection post allogeneic stem cell transplantation. *Transplant Infectious Disease*.

[8] Candoni A, Simeone E, Tiribelli M, Pipan C, Fanin R (2008). What is the optimal dosage of valganciclovir as preemptive therapy for CMV infection in allogeneic hematopoietic SCT?. *Bone Marrow Transplantation*.

[9] de la Cruz-Vicente F, Cerezuela Martinez P, Gil-Espárraga E (2008). Preemptive therapy for cytomegalovirus disease in allogeneic stem cell transplant recipients. *Transplantation Proceedings*.

[10] Wang Y, Huang XJ, Xu LP (2008). Valganciclovir for treatment of cytomegalovirus viremia in patients following allogeneic hematopoietic stem cell transplantation. *Zhonghua yi Xue za Zhi*.

[11] Takenaka K, Eto T, Nagafuji K (2009). Oral valganciclovir as preemptive therapy is effective for cytomegalovirus infection in allogeneic hematopoietic stem cell transplant recipients. *International Journal of Hematology*.

[12] Saleh AJM, Al-Mohareb F, Al-Rabiah F (2010). High efficacy and low toxicity of short-course oral valganciclovir as pre-emptive therapy for hematopoietic stem cell transplant cytomegalovirus infection. *Hematology/ Oncology and Stem Cell Therapy*.

[13] Palladino M, Laurenti L, Chiusolo P (2010). Low-dose valganciclovir as preemptive therapy for cytomegalovirus infection occurring in allogeneic stem cell transplant recipients. *Acta Haematologica*.

[14] Liu KY, Wang Y, Han MZ (2010). Valganciclovir for pre-emptive therapy of cytomegalovirus viraemia after hematopoietic stem cell transplantation: a prospective multi-center trial. *Chinese Medical Journal*.

[15] Ruiz-Camps I, Len O, de la Cámara R (2011). Valganciclovir as pre-emptive therapy for cytomegalovirus infection in allogeneic haematopoietic stem cell transplant recipients. *Antiviral Therapy*.

